# Toward time-resolved laser T-jump/X-ray probe spectroscopy in aqueous solutions

**DOI:** 10.1063/1.5129626

**Published:** 2019-12-11

**Authors:** O. Cannelli, C. Bacellar, R. A. Ingle, R. Bohinc, D. Kinschel, B. Bauer, D. S. Ferreira, D. Grolimund, G. F. Mancini, M. Chergui

**Affiliations:** 1 Laboratory of Ultrafast Spectroscopy, Lausanne Center for Ultrafast Science (LACUS), École Polytechnique Fédérale de Lausanne, CH-1015 Lausanne, Switzerland; 2 Laboratory of Femtochemistry-MicroXAS, Paul Scherrer Institut, 5232 PSI Villigen, Switzerland

## Abstract

Most chemical and biochemical reactions in nature and in industrial processes are driven by thermal effects that bring the reactants above the energy barrier for reaction. In aqueous solutions, this process can also be triggered by the laser driven temperature jump (T-jump) method, in which the water vibrational (stretch, bend, or combination) modes are excited by a short laser pulse, leading to a temperature increase in the irradiated volume within a few picoseconds. The combination of the laser T-jump with X-ray spectroscopic probes would add element-specificity as well as sensitivity to the structure, the oxidation state, and the spin state of the intermediates of reactions. Here, we present preliminary results of a near infrared pump/X-ray absorption spectroscopy probe to study the ligand exchange of an octahedral aqueous Cobalt complex, which is known to pass through intermediate steps yielding tetrahedral chlorinated as final species. The structural changes of the chemical reaction are monitored with great sensitivity, even in the presence of a mild local increase in temperature. This work opens perspectives for the study of non-light-driven reactions using time-resolved X-ray spectroscopic methods.

## INTRODUCTION

Most of the chemical and biochemical reactions that occur in solution are driven by thermal activation, which brings the systems above the barrier for reaction, as described at the end of the XIXth century by Arrhenius.^1^ The detailed study of (bio)chemical reactions has witnessed a huge leap forward with the advent of ultrafast (femtosecond to picosecond) spectroscopy,^2^ which marked the birth of Femtochemistry, allowing determination of the details of reaction pathways in a very wide variety of systems (gas phase, solutions, proteins, interfaces, etc.). In these studies, a short laser pump pulse triggers a photoinduced process (be it uni- or bimolecular) in a system whose evolution is followed by a second, short, “probe” pulse at variable time delays with respect to the pump pulse. The pump-probe method has universally been applied using pulses in the terahertz, infrared (IR), visible, and ultraviolet ranges. Over the past twenty years, it has been extended to probes such as ultrashort pulses of electrons or X-rays,^3^ via either spectroscopy or scattering and diffraction. These deliver additional insights into the evolution of the excited system by providing information about electronic and, generally not possible by optical domain methods, about nuclear degrees of freedom. Ever since the birth of Femtochemistry, only photoinitiated reactions have been studied by probing them in “real-time.” However, notwithstanding their importance in nature and applications, photoinduced processes represent only a subset of chemistry and biochemistry. It is therefore crucial to extend pump-probe spectroscopy to non-light-driven reactions and, in particular, to thermally driven ones. To this aim, a fast thermal trigger is needed to initiate the process in order to fully exploit the temporal resolution of ultrafast spectroscopy.

In the early 1950s, Manfred Eigen and co-workers inaugurated the so-called relaxation methods in chemical kinetics, which consist of impulsively perturbing a system to trigger a reaction by a temperature (T-), a pressure (P-), or a pH-jump and monitoring the return to equilibrium.^4^ The method was initially implemented in the microsecond time domain, but with the advent of pulsed lasers in the 1960s, it was pushed into the nanosecond/picosecond time domain and applied to chemical and biochemical reactions^5–10^ using optical spectroscopy, in particular of aqueous solutions.

Water is the most important solvent in nature and with the advent of ultrafast technology, short laser pulses have been utilized to induce T-jumps of 10–30 °C in aqueous media. By directly exciting the water high frequency vibrational and/or bending modes, the nonradiative relaxation of the water dissipates the energy into the bulk solution on ultrafast time scales,^11,12^ leading to an ultrafast (2–5 ps) temperature (T) increase in the irradiated volume. This impulsive T-increase can be used to locally trigger chemical reactions, whose evolutions are then monitored by optical probes.^13,14^ This innovative approach opens exciting perspectives in chemistry and functional biology, as it extends the range of applicability of ultrafast laser techniques beyond photochemistry, toward fast non-light-driven reactions.

While optical probes deliver a great degree of insight into the reactions, the identification of reactants, intermediates, and products relies on an *a-priori* knowledge of their spectroscopic features. Indeed, visible and UV spectroscopies interrogate the global electronic properties of molecular systems. Optical spectroscopic observables are the energies and intensities of valence electronic transitions, which are neither element-specific nor spin-sensitive and generally lack structural sensitivity. These limitations are, of course, transposed to time domain experiments. However, X-ray spectroscopies can overcome some of these. In particular, an X-ray absorption spectrum is characterized by edges that represent the ionization threshold of specific core orbitals (K-edge for the 1s orbital, L_1–3_ for the 2s, 2p_1/2_, and 2p_3/2_ orbitals, respectively, etc.) of the atom. At a given edge, features appear in the pre-edge region, which are due to transitions to empty or partially filled valence orbitals, providing information about the unoccupied density of states and the coordination symmetry (via the selection rules) of the investigated element. The edge itself is a sensitive probe of the element's oxidation state and its geometry, while the modulations of the absorption coefficient in the postedge region at low energies (the so-called X-ray near edge absorption structure or XANES) and at higher energies (the so-called extended X-ray absorption fine structure or EXAFS) contain information about bond lengths and angles of the coordinated surrounding atoms, i.e., they provide the local structure around the atom of interest.^3,15,16^

The past 20 years or so have witnessed the implementation of time-resolved X-ray techniques in the picosecond/femtosecond time domain at synchrotrons^15,16^ and, more recently, at X-ray free electron lasers (XFELs)^17,18^ for the study of chemical and biological systems in solution. These tools, which include X-ray absorption spectroscopy (XAS), X-ray emission spectroscopy (XES), X-ray solution scattering (XRS), and photoelectron spectroscopy (PES) of solutions,^19^ are now very well established. It therefore appears timely and even necessary to expand their use to the study of non-light-driven reactions and, specifically, to thermally driven ones. Wernet and co-workers implemented an experiment aimed at monitoring the structural changes in the hydrogen bond network of liquid water upon an impulsive T-jump heating. Specifically, they resonantly excited the intramolecular O–H stretch band of liquid water, inducing a T-jump of ∼20 °C, and monitored the transient response by oxygen K-edge absorption spectroscopy with 80 ps soft X-ray pulses from a synchrotron.^20^ This very promising study was, to our knowledge, not further expanded to the investigation of actual thermally driven chemical reactions with X-ray probes, let alone in the hard X-ray range. Notable exceptions are recent T-jump/X-ray scattering studies of protein conformational changes from the nanosecond to the millisecond time scales.^21–24^

**FIG. 1.**
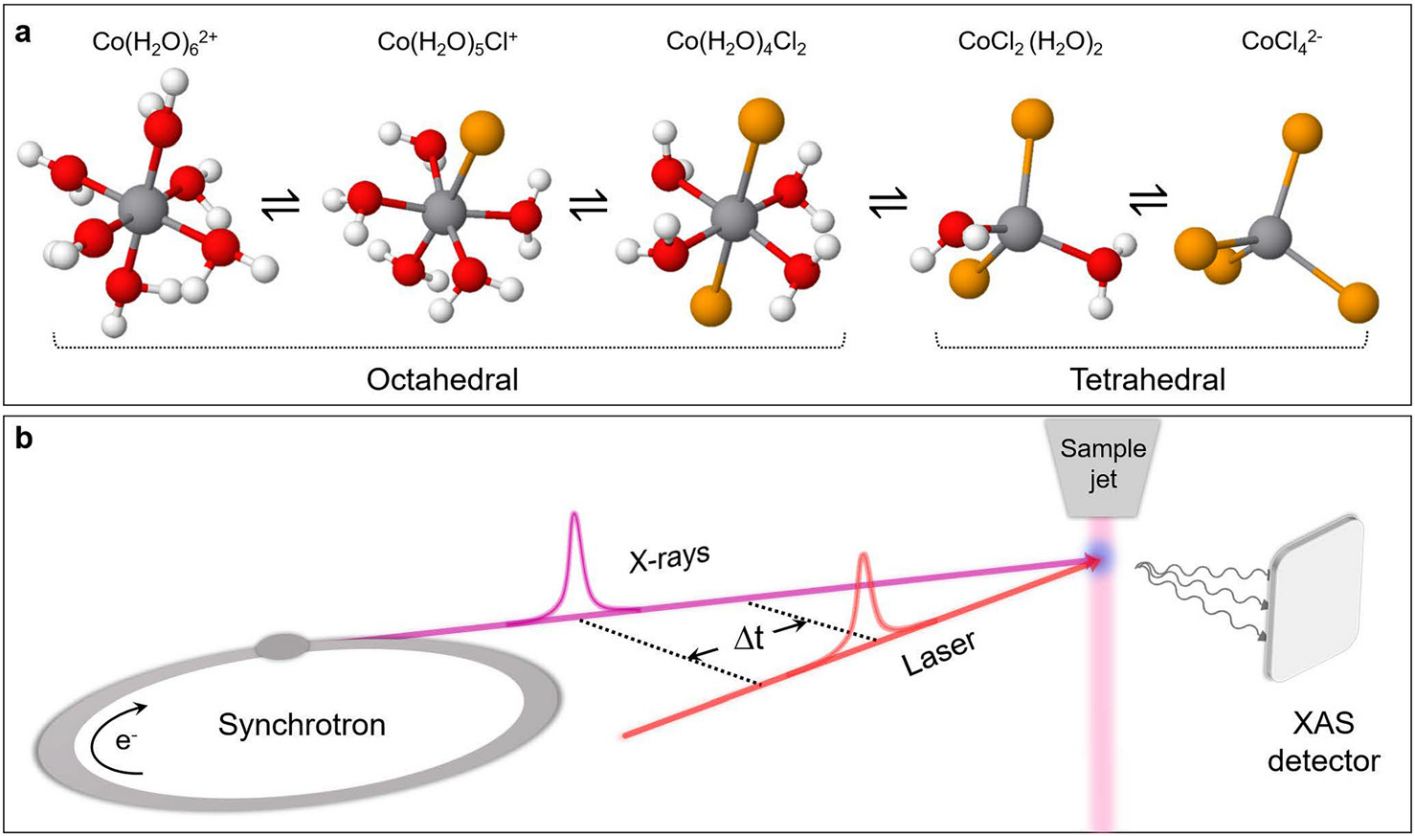
(a) Schematic layout of the investigated chemical reaction. The equilibrium can be tuned with changes in temperature or chloride concentration, causing free chloride ions to gradually replace water molecules. In parallel, structural interconversion occurs from the hexa-octahedral to chloro-tetrahedral configuration. (b) Experimental layout of the laser T-jump/X-ray absorption spectroscopy probe. The sample jet schematically represents the closed loop wire-guided liquid jet employed in the experiment.

Here, we explore the possibility of a laser T-jump/X-ray probe of a chemical reaction by monitoring a near-IR pump-induced ligand substitution reaction of a hexacoordinated Cobalt complex in a chlorinated aqueous solution by X-ray absorption spectroscopy (XAS) at the Co K-edge. At room temperature (RT), the system consists of a mixture of aquo Cobalt complexes and, upon increasing temperature and/or chloride ion concentration, can be turned into a Co-tetrachloride complex according to the following equation [see Fig. 1(a)]:

$$\text{Co}{\left(H_{2}O\right)}_{6}^{2+}+{\;4\text{Cl}}^{-}\;⇌\;{\text{CoCl}}_{4}^{2-}+{\;6H}_{2}O.$$ (1)

The choice of this model reaction is motivated by previous steady-state and time-resolved studies. The temperature dependence of the reaction had been investigated in detail using steady-state ultraviolet-visible (UV-visible) spectroscopy^25,26^ and steady-state XAS.^27,28^ The latter reported Co K-edge absorption spectra, as a function of T and/or chloride concentration ([Cl^−^]). The authors identified the spectral signatures of the octahedral and tetrahedral species, along with those of the intermediates, establishing the reaction sequence [Fig. 1(a)]. Ma *et al.*^29^ performed an ultrafast T-jump/optical probe study of the reaction exciting the overtone stretch mode of water at 1.55 *μ*m. Three rate constants were reported, and the analysis of their T-dependence was interpreted as supportive of the associative interchange reaction mechanism. The insights into the reaction kinetics and energetics provided by this study, along with the above-mentioned steady-state investigations, serve as a basis for a proof-of-principle of the corresponding X-ray spectroscopic experiment.

Here, we explore the possibility of laser T-jump experiments in combination with an X-ray absorption spectroscopy (XAS) probe. Our experiment uses a pump wavelength of 1.064 *μ*m. At this wavelength, not only the overtone vibrations of water but also of the dipole-forbidden d-d transition of the [Co(H_2_O)_6_]^2+^ complex are excited (see below). Although this may not represent a pure T-jump, we argue how our results provide a basis for future T-jump/X-ray probe experiments of chemical reactions. We also discuss novel perspectives for the investigation of T-activated chemical and biochemical processes using a broad range of X-ray spectroscopic (absorption, emission, and photoelectron spectroscopies) and scattering (elastic and inelastic) techniques.

## EXPERIMENTAL METHODS

The measurements were carried out at the MicroXAS beamline of the Swiss Light Source (SLS) (Paul Scherrer Institute, Villigen). The sample consists of a water solution of [Co^2+^] = 500 mM (CoCl_2_ hexahydrate salt, abcr, purity 99.9%) and [Cl^−^] = 8 M (LiCl salt, Roth, purity 99% min.), which also accounts for the Chlorine contribution coming from the CoCl_2_ salt. A closed loop wire-guided liquid jet (250 *μ*m thickness)^30^ was employed as a sample delivery system. The solution temperature was kept constant by means of a thermostat.

The experimental layout is schematized in Fig. 1(b). Transient XAS measurements were performed using the isolated hybrid X-ray pulse provided by the Swiss Light Source (SLS) synchrotron, with a time duration of 70 ps, to probe both the Co K-edge XANES and EXAFS regions. A high repetition-rate Duetto laser (10 ps pulses at 1064 nm fundamental wavelength, 32 *μ*J pulse energy) was utilized to excite the solution by pumping a combination mode of high-frequency overtones of the symmetric and antisymmetric stretching vibrations of water^11^ and the d-d transition of the octahedral Cobalt complexes, in a near-collinear geometry to the X-ray beam. The data acquisition system is described in Ref. 31. We used the total fluorescence yield detection, with avalanche photodiodes in orthogonal geometry with respect to the X-ray beam. The X-ray signal was acquired on a pump-on/pump-off basis at 130 kHz, with the laser at 65 kHz in order to increase the energy per pulse deposited into the irradiated volume.

The jet speed (6 m/s) and the laser and X-rays spot sizes of 70 × 60 *μ*m^2^ and 50 × 50 *μ*m^2^ Full Width at Half Maximum (FWHM), respectively, were chosen to avoid multiple pumping of the same spot at the sample position. Reference steady-state X-ray absorption spectra were also recorded at the Co K-edge (7.700–7.800 keV, 2 eV step-size), using a Ketek fluorescence detector. Reagent and product spectra were respectively collected for a [Co(H_2_O)_6_^2+^] = 250 mM water solution at room temperature (CoSO_4_, Roth, purity 99% min.) and a [CoCl_4_][N(CH_3_)_4_]_2(s)_ pellet (Sigma-Aldrich, purity 99.5% min.), a solid standard containing CoCl_4_^2−^ groups. In our experiment, the starting temperature and solute and salt concentrations T = 60 °C, [Co^2+^] = 500 mM, and [Cl^−^] = 8 M, respectively, were selected to demonstrate the sensitivity of the near-IR pumping scheme even in the extreme case of the weak absorption regime from the solvent, yet maximizing the signal-to-noise ratio.

## RESULTS

**FIG. 2.**
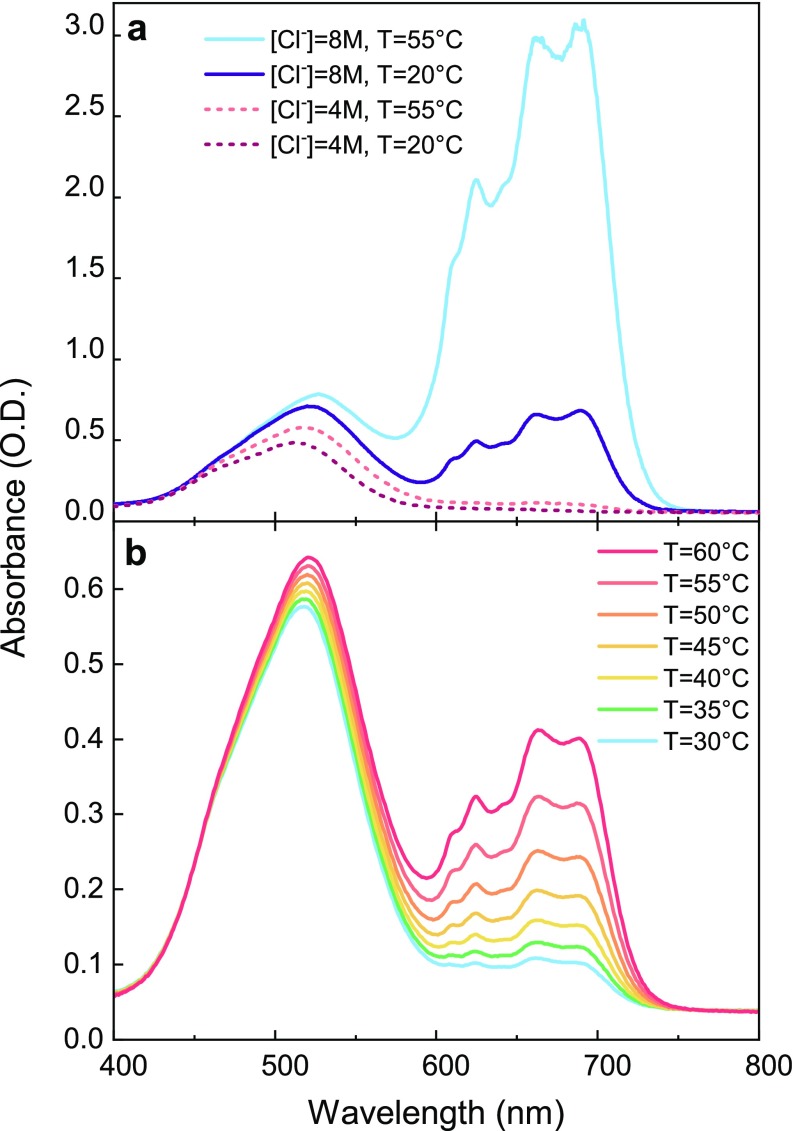
UV-visible spectra for a [Co^2+^] = 1 M solution. (a) Spectra at two different temperatures, T = 20 °C and T = 55 °C, [Cl^−^] = 4 M (dashed curves) and [Cl^−^] = 8 M (solid curves). (b) The effects of the temperature in the range of 30–60 °C for a [Cl^−^] = 7 M solution. The strong increase in the 600–750 nm bands is associated with the tetrahedral product formation, while the redshift of the 500 nm band is related to the formation of octahedral chlorinated intermediates.

In the chemical reaction under investigation [Fig. 1(a)], the geometry of each intermediate is dictated by the crystal field stabilization energy,^27,32,33^ implying an octahedral configuration for the first three structures and a tetrahedral configuration for the last two. The equilibrium constant for the overall process is

$$K_{\mathrm{eq}}(T)=\frac{\left[{\mathrm{CoCl}}_{4}^{2-}\right]}{[{\mathrm{Co}(H_{2}O)}_{6}^{2+}]{\left[{\mathrm{Cl}}^{-}\right]}^{4}},$$ (2)

which indicates that the reaction is sensitive to the ligand concentration. Either an increase in the chloride ion concentration [Cl^−^] or an increase in temperature, the reaction being endergonic, promotes the gradual replacement of the coordinated water molecules by chloride ions, thereby moving the equilibrium in favor of the tetrahedral products. The determination of the [Co^2+^], [Cl^−^], and T parameters, which maximize both the XAS signal and the thermally induced product formation, is key to the success of a T-jump experiment. The X-ray signal is proportional to the number of Cobalt centers dissolved in the solution. However, a high [Cl^−^]/[Co^2+^] is needed in order to move the equilibrium toward the product. Under this constraint, the upper limit to [Co^2+^] is actually set by the solubility of LiCl in water. Therefore, a [Co^2+^] = 500 mM was chosen as a compromise, yielding a strong XAS signal while keeping the possibility of a high [Cl^−^]/[Co^2+^] ratio. Since the conversion process is accompanied by a color change of the solution due to the characteristic absorption spectra of reagent and product, the optimal [Cl^−^] and T values were determined using static UV-visible spectroscopy, as seen in Figs. 2(a) and 2(b).

In the literature, the absorption spectra have already been discussed in terms of ligand field theory^25,34^ and a detailed assignment of the bands was recently performed.^26^ The broad band centered around 500 nm is associated with two weak Laporte-forbidden d-d electronic transitions of the octahedral species, whereas the feature in the 600–750 nm region is due to a strong electronic transitions of the tetrahedral complexes. This region exhibits a partially resolved fine structure, probably due to spin–orbit coupling and/or vibronic coupling.^25^

**FIG. 3.**
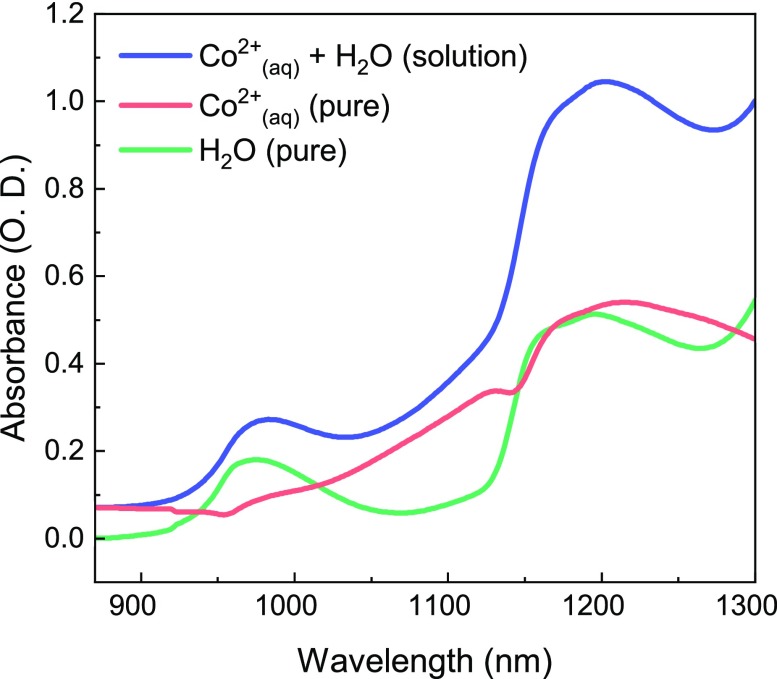
Static UV-visible reference spectra of the [Co^2+^] = 500 mM, [Cl^−^] = 8 M water solution (blue trace), compared to pure water (green trace) and the pure Cobalt contribution (red trace, taken from the difference between the blue and the green traces). A common offset was subtracted from both the spectra, corresponding to the intensity of the pure water solution at 860 nm, where, according to Ref. 35, the absorbance of the water is almost negligible.

Figure 2(a) shows the UV-visible spectra for a [Co^2+^] = 1 M solution at two different temperatures, T = 20 °C and T = 55 °C, and for two different chloride concentrations, [Cl^−^] = 4 M and [Cl^−^] = 8 M. The presence of the 500 nm and 600–750 nm bands confirms the copresence of reagents, intermediates, and products under equilibrium conditions. Upon a temperature increase, a partial conversion from the hexa-aquo complex to the octahedral chlorinated intermediates and the tetrachloride complex occurs. The band centered at 510 nm increases and shifts toward the red, a behavior associated with the partial conversion of the reagent into octahedral chlorinated species,^26^ while the intense 600–750 nm band increases in intensity due to the product formation. A similar effect occurs upon increasing the chloride concentration from 4 to 8 M, which moves the equilibrium toward the products. The temperature effect is about 2.5 stronger for the [Cl^−^] = 8 M solution, meaning the larger the chloride concentration, the higher the product formation and the expected transient signal. The T-effect on the UV-visible spectra in the 30–60 °C range and at a constant [Cl^−^] concentration is shown in Fig. 2(b) for a [Co^2+^] = 1 M and [Cl^−^] = 7 M water solution. For the same temperature change of 5 °C, the increase in the 600–750 nm product bands is more pronounced at higher temperature. Consequently, the experimental conditions that give a strong XAS signal and a strong temperature-induced complex conversion are high [Co^2+^] and [Cl^−^] concentrations and an elevated T. This set of parameters is consistent with those reported by Ma *et al.* in their T-jump/UV-visible probe study.^29^ The octahedral complexes also have a weak and broad band, which is centered at 1250 nm and has a width that covers a range from 1000 to 1600 nm, as seen in Fig. 3. This band is assigned to a d-d transition from a nonbonding t_2g_ to an antibonding e_g_^*^ orbital. We remark that, although this band contributes significantly to the absorption in the region of our pump wavelength, under the assumptions we explain below, we believe the conclusions constitute evidence for a T-jump experiment probed with XAS.

**FIG. 4.**
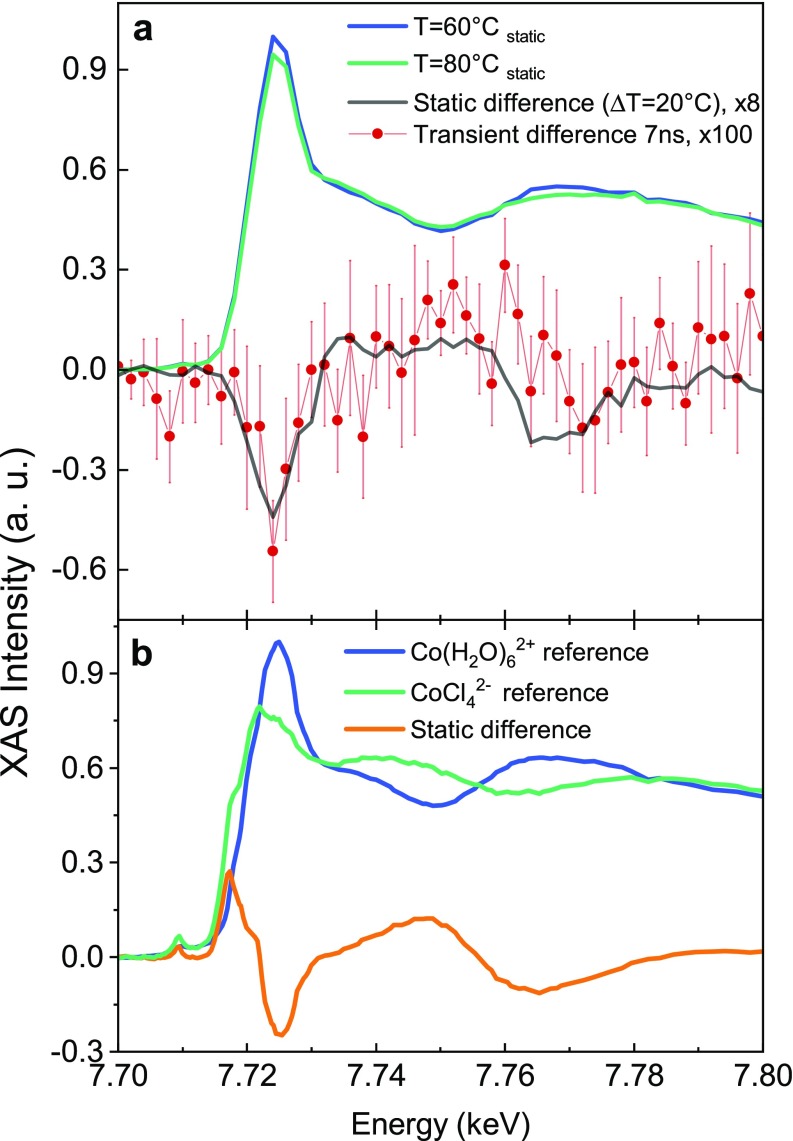
Laser T-jump/XAS probe study on a [Co^2+^] = 500 mM, [Cl^−^] = 8 M water solution. (a) Static XAS spectra were taken without the laser at 80 °C (green) and 60 °C (blue). The difference between the two static XAS spectra at T = 80 °C and T = 60 °C is displayed by the black trace (scaled by ×8) and overlaid with the pump-probe data (scaled by ×100) obtained at 7 ns by the laser-induced T-jump (red dots). The static spectra were scaled with a common factor (maximum of the T = 60 °C curve), keeping the presence of the isosbestic points. (b) Reference static X-ray absorption spectra at the Co K-edge. Blue: [Co(H_2_O)_6_^2+^] = 250 mM water solution at room temperature. Green: [CoCl_4_][N(CH_3_)_4_]_2(s)_ pellet. Their static difference is displayed by the orange solid curve. The spectra were scaled in order to have the same integrated area below the curves. In this way, the presence of the isosbestic points is retrieved.

We recorded the steady-state XAS spectra at 60 and 80 °C in order to determine the X-ray spectroscopic changes expected under a significant temperature increase [Fig. 4(a)]. The XAS spectra exhibit a prominent white line at the edge at 7.725 keV, associated with the 1s → 4p Cobalt core transition, whose intensity decreases with temperature. Postedge intensity changes are observed around two isosbestic points at 7.737 and 7.757 keV, in agreement with the results of Liu *et al.*^27^ We therefore set the sample aqueous solution ([Co^2+^] = 500 mM, [Cl^−^] = 8 M) to T = 60 °C and utilized the 1064 nm laser light to pump the system, which was probed by XAS at a 7 ns time delay. The choice of the time-delay is based on the results of Ref. 29, which showed that, after 4 ns, the concentrations of reagents and products have reached a plateau and the equilibrium associated with the new temperature is established. Thus, both the 7 ns pump-probe transient and the difference of steady-state XAS correspond to the subtraction of two spectra that reflect the distribution of Cobalt complexes equilibrated at two different temperatures. The same results are compared, bearing in mind that the transient spectrum shows a laser induced effect and not a difference of steady-state spectra. Figure 4(a) shows the transient XAS signal at 7 ns (red dots) and the difference of the steady-state XAS spectra at 80 and 60 °C (black solid line), which are in excellent agreement.

**FIG. 5.**
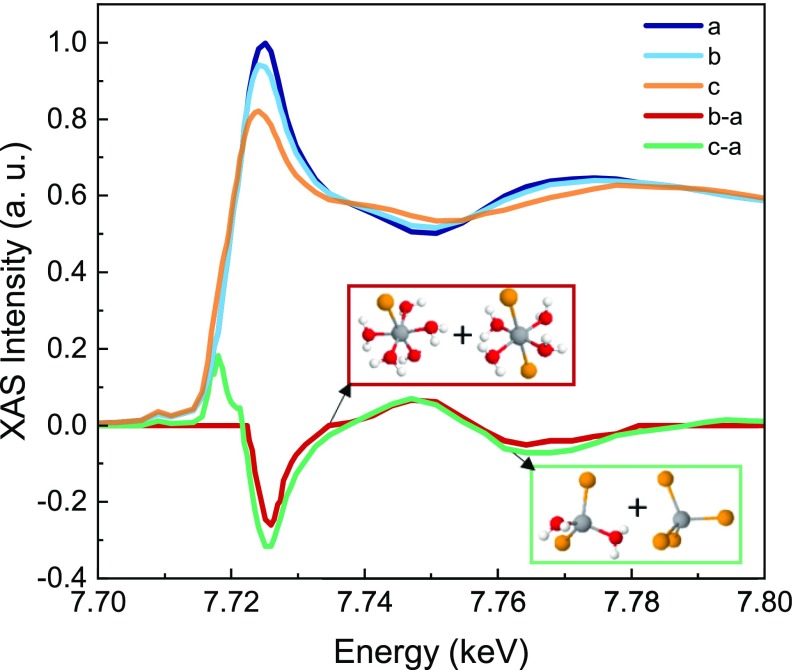
Assignment of spectral features to reaction intermediates. All traces were digitized as is from Ref. 27. In this panel, (a) NaCl = 0 M, 250 °C; (b) NaCl = 0.5 M, 250 °C; (c) NaCl = 1.5 M, 250 °C. The red solid line corresponds to (b-a) (scaled ×4), and it is ascribed to the octahedral compound mixture formation. The green solid line corresponds to (c-a), and it is ascribed to the tetrahedral compound mixture formation at the expenses of the reagent.

The interpretation of the transient signal requires the knowledge of the XAS features as the structural fingerprint of the Cobalt complexes. To this aim, we measured the spectra of the reference compounds corresponding to the start Co(H_2_O)_6_^2+^_(aq)_ and end CoCl_4_^2−^_(s)_ species of the reaction. These spectra are shown in Fig. 4(b), along with their static difference. The latter shows an intensity drop around the main peak at 7.725 keV (the so-called white line) and the appearance of postedge modulations. Both the intensity drop of the white line and the postedge modulations are reminiscent of those observed in the 7 ns transient [Fig. 4(a)]. However, two additional features appear in the static difference of Fig. 4(b): a small pre-edge peak related to 1s → 3d Cobalt electronic transitions and an intense shoulder in the 7.716–7.720 keV range. To rationalize these differences, we compare our spectra with those of Liu *et al.* in Ref. 27, which we have digitized and show in Fig. 5. These authors reported a systematic investigation of chloride concentration and temperature effects on the steady-state Co K-edge absorption spectra for an aqueous Cobalt(II) chloride solution. Specifically, they identified the XAS spectra of the reagents and the mixture of the two tetrahedral products through a principal component analysis, i.e., a dataset reduction of the recorded XAS spectra. Their XAS analysis shows that the decrease in the white line intensity is spectrally related to the conversion of the reagent into the octahedral chlorinated intermediates (red trace), whereas the presence of the shoulder and the main edge depletion is ascribed to the formation of Cobalt chloride tetrahedral species (green trace), also consistent with the conclusions of a recent *in situ* XAS investigation.^28^

The agreement between our transient data at 7 ns [Fig. 4(a)] and the static difference for the reagent-octahedral intermediate mixture (Fig. 5) suggests that, in the present experiment, the excitation drove the equilibrium toward a mixture of the octahedral intermediate species, Co(H_2_O)_5_Cl^+^ or Co(H_2_O)_4_Cl_2_, without full structural conversion to tetrahedral complexes.

## DISCUSSION

Assuming all absorbed photons end up heating the irradiated volume, the peak temperature jump can be estimated following Wang *et al.*,^12^

$$\Delta T=\frac{2E\alpha }{A\rho C_{v}},$$ (3)

where E is the laser pulse energy (32 *μ*J), α is the absorption coefficient of the solution at 1064 nm, A is the laser spot area at the focus, ρ is the water density, and C_v_ is the water heat capacity at room temperature. In our experimental conditions, ΔT corresponds to a mild increase in 0.13 °C. As a comparison, previously reported T-jump experiments pumped the water absorption peaks located at either 3400 cm^−1^ or 6600 cm^−1^, where the absorption coefficient is ∼10^2^–10^4^ stronger.^35^ Under these conditions, ΔTs of 20–40 °C (Ref. 20) or 5–10 °C (Ref. 29) were reached using very high fluences. As previously mentioned, the absorption at 1064 nm is due to both water and the hexacoordinated complexes (Fig. 3). Our estimate of the absorption coefficient α at 1064 nm accounts for both the contribution from the water, α = 0.06 cm^−1^, and that of Co(H_2_O)_6_^2+^, for which α = 0.21 cm^−1^ with [Co^2+^] = 500 mM. Since most of the absorption at 1064 nm is due to the d-d transition^25^ of the complexes, one may question whether the observed effects are really due to thermal effects or are caused by photodissociation of an H_2_O ligand followed by association Cl^−^ one.

In the event where there is no photodissociation of octahedral Cobalt complexes, it is most likely that the intramolecular relaxation of the excited complex will lead to heat release to the environment. A good example is provided by the case of aqueous [Fe(CN)_6_]^3+^, where the heating of water was found to be on the same time scale (few picoseconds) as the vibrational cooling of the nitrogen cyanide (CN) stretch frequencies.^36^ In addition, a long-lived excited state can reasonably be excluded based on the results of pump-probe experiments of octahedral Co(III) or Co(II) complexes, which show that the relaxation of the systems is complete in few picoseconds.^37,38^

In the event of photodissociation of octahedral Cobalt complexes and considering that, at the present concentrations, there are 16 Cl atoms and 111 water molecules per octahedral complex, the probability of associating a Cl^−^ ion with the pentacoordinated fragment is not negligible. However, these events generally take place at much shorter time scales than the nanosecond regime. For example, in the case of aqueous [Fe(CN)_6_]^4+^, the uptake of a water molecule after dissociation of a CN fragment requires ∼20 ps.^39^ In the case of hexacarbonyl metals such as Cr, W, and Mo, association times were on the few picoseconds time scale.^40^ Finally, in the case of aqueous cis-[Ru(bpy)_2_(CH_3_CN)_2_]Cl_2_, the substitution of one of the CH_3_CN ligands by a water molecule requires ∼80 ps.^41^ Accounting for the hypothetical presence of chlorinated species generated with photodissociation of the reagent, their contribution to a transient signal would be short-lived. Indeed, in the absence of a temperature change, the thermal reaction would be responsible for re-establishing the equilibrium distribution of the Co complexes dictated by the starting temperature, fulfilling the conditions imposed by Eq. (2) and washing out any out-of-equilibrium photoproducts. Provided the chemical reaction is investigated at longer time scales than the time required for the thermal reaction to occur, no photoproducts will be probed. The T-jump optical-domain investigation of the same reaction^29^ showed that the thermally induced product formation is complete in about 4 ns. Therefore, based on simple kinetics and thermodynamic arguments, we can safely exclude any direct photoinduced product formation, via dissociation-association, to the observed transient signal at the long time delays considered here (7 ns).

**FIG. 6.**
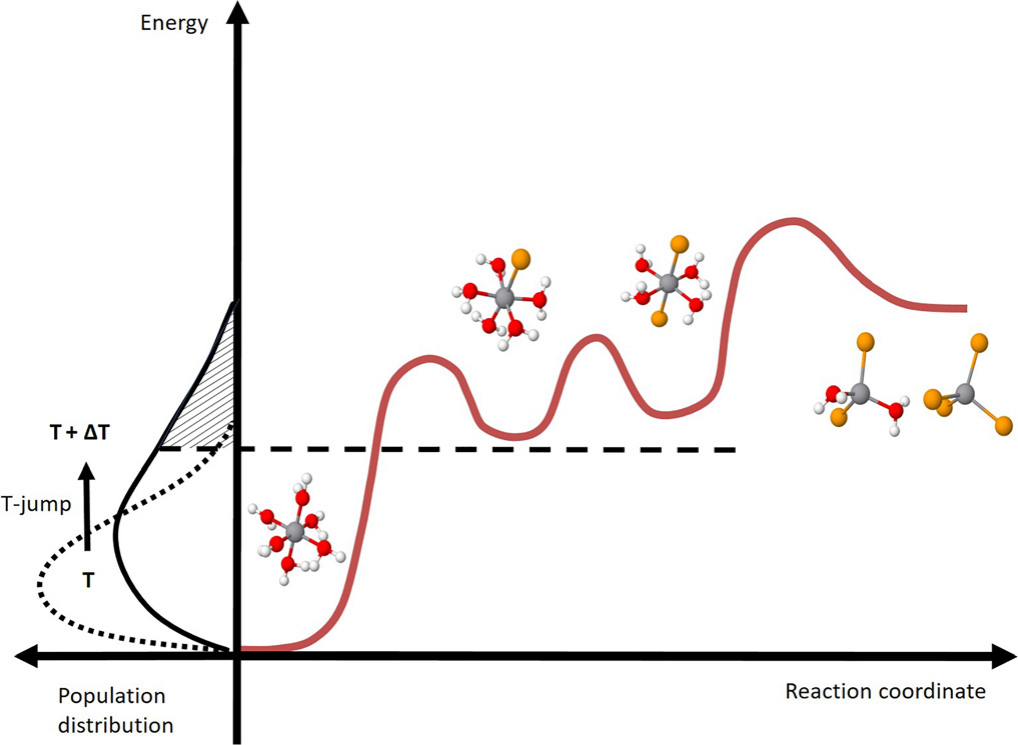
Schematic of the energy landscape as a function of the reaction coordinate for the investigated chemical equilibrium: changes of the energy distribution of the complexes as a function of temperature (left) and related conversion of the reagent into intermediates and products (right).

Based on these premises, the laser induced effect can be interpreted as a genuine T-jump at 7 ns. The above results allow us to elaborate more on the T-jump approach in general and on its difference with respect to static (*in situ*) investigation. *In situ* methods change the steady-state, average distribution of the species involved in the chemical equilibrium.^27,28^ The T-jump instead locally triggers a chemical equilibrium away from the reagents, and it acts as a tool to isolate—at specific time delays—the effect of the ongoing reaction with respect to the initial conditions, eventually reaching the equilibrium associated with the new temperature and converging to the steady state results. In a general picture, the amplitude of the sudden temperature increase impacts on the speed of the reaction rate (Arrhenius law) and on the number of molecules involved in the chemical transformation. The former determines which intermediates/products will be probed, and the latter determines how strong the transient signal will be. In our experiment, the XAS spectrum associated with a mild T-jump of 0.13 °C shows the same profile as the difference of steady state spectra being 20 °C apart [Fig. 4(a)]. This implies that the same octahedral intermediates are produced at the expense of the reagent. However, the amount of the reagents converted in these two cases is different, leading to transients of significantly different intensities. This concept is schematized in Fig. 6: upon a 0.13 °C T-jump, the distribution of energy changes among the complexes and, within 7 ns, the chemical equilibrium at the higher temperature is established. This includes a larger amount of octahedral intermediates and, probably, a minor population of tetrahedral products whose signals are not sufficient to overcome the noise of our measurement.

This scheme allows us to suggest a reaction landscape where the reagents are thermodynamically more stable than the octahedral intermediates that, in turn, are more stable than the tetrahedral products (Fig. 6). No kinetic information is retrieved without the time evolution of the concentration of the species, representing the final goal of the investigation of the reaction mechanism. In this respect, XAS represents a powerful complementary approach to the UV-visible spectroscopy of a T-jump induced process. In the optical domain, the CoCl_4_^2–^ absorption band is strong enough to allow the detection of products, even under experimental conditions where their concentration is almost negligible. Instead, X-ray probing is sensitive to the relative weight of all chemical species, pointing to a large majority of octahedral complexes and providing structural information about the elusive reaction intermediates.

## CONCLUSION AND OUTLOOK

The present study is a first step toward implementing X-ray probing of fast thermally induced chemical reactions in solutions. By pumping an aqueous solution containing hexacoordinated Co^2+^ ions in the presence of a high concentration of Cl^−^ ions using a 10 ps pulse at 1064 nm, we have driven, up to the octahedral chlorinated intermediates, the reaction that ultimately leads to a tetrachloro-complex. The products were detected by X-ray absorption spectroscopy using 70 ps X-ray pulses from a synchrotron. The exquisite sensitivity to the chemical composition and structure makes X-ray spectroscopy ideal to observe chemical and biochemical reactions under their most common and relevant conditions. The present work calls for further studies to fully benefit from the X-ray probing of thermally induced reactions. First, the choice of the laser pump wavelength should be tuned to the red, typically around 1.5 *μ*m, in the overtone of the water stretch mode, in order to heat the irradiated volume more efficiently and thus increase both the transient signal and the reaction rate, avoiding any direct excitation of the educt. Second, because of the ultrafast (2–5 ps) heating of liquid water, extending the method to shorter time scales, i.e., faster thermally driven reaction, is now possible, using synchrotron pulses (∼70 ps) or even X-ray free electron laser pulses (<100 fs). Third, the implementation of the T-jump method with X-ray probing is by no means limited to X-ray absorption spectroscopy but can be extended to solution X-ray scattering, X-ray emission spectroscopy, and liquid phase photoelectron spectroscopy. In particular, the latter two should help distinguish the various intermediates in terms of speciation, while the structural information is derived from XAS. Furthermore, with the development of new delivery schemes of solutions into vacuum,^42,43^ the extension of T-jump induced reactions using soft X-ray probing is now possible, with the advantage that light elements such as N, C, O, and F, can be selectively detected, opening perspectives to study organic and biochemical reactions in solution. We believe the present results represent an opportunity to extend the investigation of chemical reactions at synchrotron and X-ray free electron lasers beyond photoinduced ones into temperature-driven (bio)chemical processes.
